# MicroRNA-940 as a Potential Serum Biomarker for Prostate Cancer

**DOI:** 10.3389/fonc.2021.628094

**Published:** 2021-03-19

**Authors:** Smrithi Rajendiran, Sayantan Maji, Ahmed Haddad, Yair Lotan, Rajesh R. Nandy, Jamboor K. Vishwanatha, Pankaj Chaudhary

**Affiliations:** ^1^Department of Microbiology, Immunology, and Genetics, University of North Texas Health Science Center, Fort Worth, TX, United States; ^2^Department of Urology, University of Texas Southwestern Medical Center, Dallas, TX, United States; ^3^School of Public Health, University of North Texas Health Science Center, Fort Worth, TX, United States; ^4^Texas Center for Health Disparities, University of North Texas Health Science Center, Fort Worth, TX, United States

**Keywords:** prostate cancer, miR-940, serum, circulating miRNA, PSA

## Abstract

Prostate cancer is one of the leading causes of death despite an astoundingly high survival rate for localized tumors. Though prostate specific antigen (PSA) test, performed in conjunction with digital rectal examinations, is reasonably accurate, there are major caveats requiring a thorough assessment of risks and benefits prior to conducting the test. MicroRNAs, a class of small non-coding RNAs, are stable molecules that can be detected in circulation by non-invasive methods and have gained importance in cancer prognosis and diagnosis in the recent years. Here, we investigate circulating miR-940, a miRNA known to play a role in prostate cancer progression, in both cell culture supernatants as well as patient serum and urine samples to determine the utility of miR-940 as a new molecular marker for prostate cancer detection. We found that miR-940 was significantly higher in serum from cancer patients, specifically those with clinically significant tumors (GS ≥ 7). Analysis of receiver operating characteristic curve demonstrated that miR-940 in combination with PSA had a higher area under curve value (AUC: 0.818) than the miR-940 alone (AUC: 0.75) for the diagnosis of prostate cancer. This study provides promising results suggesting the use of miR-940 for prostate cancer diagnosis.

## Introduction

Prostate cancer is one of the leading causes of cancer related death among men in the US despite the advances in biomarkers (1). About 20% of all estimated new cases and 10% of estimated deaths in men are expected to be due to prostate cancer (2). Though many prostate cancers are slow growing, some forms are very aggressive and metastatic, resulting in death. Depending on the racial background and family history, some men are advised to undergo digital rectal exam (DRE) and serum total prostate specific antigen (PSA) screening at as early as 40 years of age. Some people inherently have high levels of PSA as a result of enlarged prostate, resulting in extensive evaluation with repeat biopsies. Most patients with abnormal PSA have insignificant prostate cancer, thus PSA screening has the potential for over-diagnosis and unnecessary treatments (3). Additionally, a report by U.S. Preventive Services Task Force recommended against PSA-based screening unless men express a preference for screening after being informed of and understanding the benefits and risks (4–6). Though prostate cancer antigen 3 (PCA3) urine test is under consideration as a better prognostic marker, there is insufficient evidence of extensive improvements in sensitivity and specificity compared to PSA (7, 8). Additionally, though the test marginally improves the diagnostic accuracy of prostate cancer, it does not predict short-term or long-term outcomes or the need for biopsies (7, 9–11). Further, the PCA3 test does not necessarily help distinguish aggressive from indolent disease (12, 13). Thus, there is a need for a more reliable and cost-effective tool for prostate cancer screening.

MicroRNAs (miRNAs) are a class of small non-coding RNA that regulate gene expression post-transcriptionally (14, 15). Various studies report miRNAs to be highly deregulated in prostate cancer (14, 16, 17). Additionally, miRNAs are highly stable in the serum, providing potential for using these circulating molecules as molecular diagnostic markers of cancer (17–21). The miRNA signatures are also helpful in determination of the primary site in tumors of unknown origin (22–24). Use of the miRNAs as blood-based markers has tremendous potential when used in combination with the existing PSA screening techniques for the status of the disease—cancer or not (25–27), and course of treatment (28–31).

We have identified miR-940, expressed on chromosome 16 in humans, as a novel miRNA that impedes prostate cancer progression in an *in vitro* system by regulating MIEN1 (32). Various other groups have shown dysregulation of miR-940 in other cancers. In hepatocellular carcinoma, glioma, non-small cell lung carcinoma, pancreatic ductal adenocarcinoma, triple negative breast cancer, and ovarian cancer, miR-940 inhibits cancer progression (33–39); while the opposite effects are observed in endometrial and cervical cancers (40–42).

Here, we aim to determine if circulating miR-940 could be used for early detection of prostate cancer and to distinguish between the clinically significant (GS ≥ 7) and insignificant (GS = 6) tumors, leading to improved management. Analysis of miR-940 in the prostate cancer and immortalized normal cell culture supernatants and serum samples from normal donors and cancer patients provides promise for considering miR-940 as a potential biomarker for prostate cancer. MiR-940 distinguishes between clinically insignificant and significant tumors, but owing to the small sample cohorts in the various groups, the statistical significance we observed is not appreciably better than the existing test (PSA) characteristics (43). Interestingly, we were also able to detect miR-940 in the urine of patients with cancer of GS ≥ 6; but this was at much lower levels than in the serum and was not different between clinically insignificant and clinically significant tumor samples suggesting a higher probability of success in prostate cancer diagnosis when using serum for miR-940 detection.

## Materials and Methods

### Cell Lines and Cell Culture

Human prostate carcinoma cells DU-145 (ATCC HTB-81) was maintained in RPMI 1640 media with 10% fetal bovine serum (Life Technologies). Immortalized non-tumorigenic prostate epithelial cell line PWR-1E (ATCC CRL-11611) was maintained in Keratinocyte-SFM (Life Technologies) supplemented with bovine pituitary extract (25 μg/ml) and recombinant epidermal growth factor (0.15 ng/ml). Cells were cultured at 37°C with 5% CO_2_.

### Exosome Isolation From Cell Culture Supernatant

Exosomes from cell culture supernatant were isolated as described previously (44). Briefly, the cells were grown in serum free medium for at least 24 h. The conditioned medium was transferred to conical tubes and centrifuged at 300 × g for 10 min at 4°C to pellet the cells. Next, the supernatant was transferred to ultracentrifuge tubes and centrifuged at 16,500 × g for 20 min at 4°C to further remove cells and cell debris. The supernatant was passed through a 0.2 μm filter to remove particles larger than 200 nm. Next, the filtered supernatant was transferred to new ultracentrifuge tubes and centrifuged at 120,000 × g for 70 min at 4°C to pellet the exosomes. The supernatant was discarded and resuspended in TRIzol and processed for RNA isolation.

### Serum and Urine Sample Collection From Normal and Prostate Cancer Patients

Serum samples (*n* = 32) from prostate cancer patients who had not undergone any treatment at the time of sample collection were obtained by Dr. Yair Lotan between 2001 and 2010 and scored based on pathology scoring criteria at the time of collection. Of this pool, the pathological Gleason Score was ≥ 7 in 25 patients and Gleason Score 6 in 7 patients. Normal serum samples (*n* = 25) was collected by the University of North Texas Health Science Center, Fort Worth, TX, through a collaboration with Dallas Methodist Hospital at Dallas, TX, during prostate cancer education and screening program from healthy males, who at the time of sample collection had no obvious indication of prostate cancer. The collection process was the standard blood draw procedure, performed by a well-trained phlebotomist at the respective sites. The urine samples were collected from patients at the time of their biopsy by Dr. Yair Lotan in 2015. Subjects were consented to the study and no personal health information was revealed. In short, for serum collection, venous blood was collected from each patient and centrifuged. Similarly, for urine miRNA, urine was collected and centrifuged. The supernatants (serum and urine) were recovered, aliquoted and stored at −80°C until further use. The study protocol was approved by the Institutional Review Boards of the University of North Texas Health Science Center, Fort Worth, TX (IRB # 2007-110, and 2013-016) and the University of Texas Southwestern Medical Center, Dallas, TX (IRB # STU 032011-187).

### RNA Isolation and qPCR

Total RNA was isolated from the exosomes collected using TRIzol (Life Technologies) according to the standard protocol. Total RNA from the serum was isolated using the miRVANA PARIS kit (Life Technologies), according to the instructions provided by manufacturer. Equal amount of quantified RNA was used for the first step of cDNA synthesis using NCode VILO miRNA cDNA Synthesis Kit (Life Technologies). For the expression of the miRNA, first the miRNA specific forward primers (mature and precursor) and normalization control specific forward primers were designed as described by Kramer 2011 (45) using Primer 3 (46) software and synthesized by Integrated DNA Technologies (Coralville, IA). The specificity of both mature and precursor primers were further confirmed by NCBI BLAST software (47). Next, using EXPRESS SYBR GreenER miRNA qRT-PCR Kits (Life Technologies), the qPCR for the miRNA and controls were carried out, according to the protocol provided by manufacturer on a Mastercycler ep gradient S realplex^2^ thermal cycler (Eppendorf). Normalization of samples was carried out with respect to RNU6-2 expression. The average of the ΔCt values of all the normal samples was used to obtain the ΔΔCt values for each individual sample (normal and cancer). This ΔΔCt was then used to determine the fold change (the normalized expression values). For urine analysis, equal amounts of urine samples (625 μL per sample) was used to isolate the total RNA using miRVANA PARIS Kit, according to the manufacturer's protocol. In brief, we measured and transferred the aqueous phase, post-phenol-chloroform centrifugation, into two tubes, equally. Then, one of the tubes was precipitated with 1.25 volumes of room temperature 100% ethanol to extract total RNA from the sample and the other tube with 0.33 volume of room temperature 100% ethanol to extract enriched small RNAs. The qPCR analysis was performed as described above. ΔCt values for qPCR were obtained as Ct of miR-940 in enriched fraction—Ct of miR-940 in total RNA fraction and then compared to the averages obtained from the clinically insignificant (GS = 6) samples.

### Statistical Analyses

Column graphs were generated as mean ± standard error of means (SEM) where applicable. The Scatter plots were generated as mean ± SEM. and *p*-value was calculated according to unpaired *t*-test with or without Welch's correction according to the distribution using GraphPad Prism 8 (GraphPad Software, CA). Receiver operating characteristic (ROC) curve and area under the curve (AUC) value were generated based on the sensitivity and the specificity of the different data points by the GraphPad Prism 8. Sensitivity and the specificity values for the ROC curves with individual predictors (PSA or miR-940) are calculated empirically. However, for combined predictors (PSA and miR-940), sensitivity and the specificity values cannot be obtained empirically. Instead, these values were calculated by fitting a logistic regression model with PSA and miR-940 as predictors. Spearman correlation coefficient between PSA and miR-940 was also calculated in GraphPad Prism.

## Results

### Prostate Cancer Cells Secrete More miR-940 Than Normal Cells

**Figure 1:**
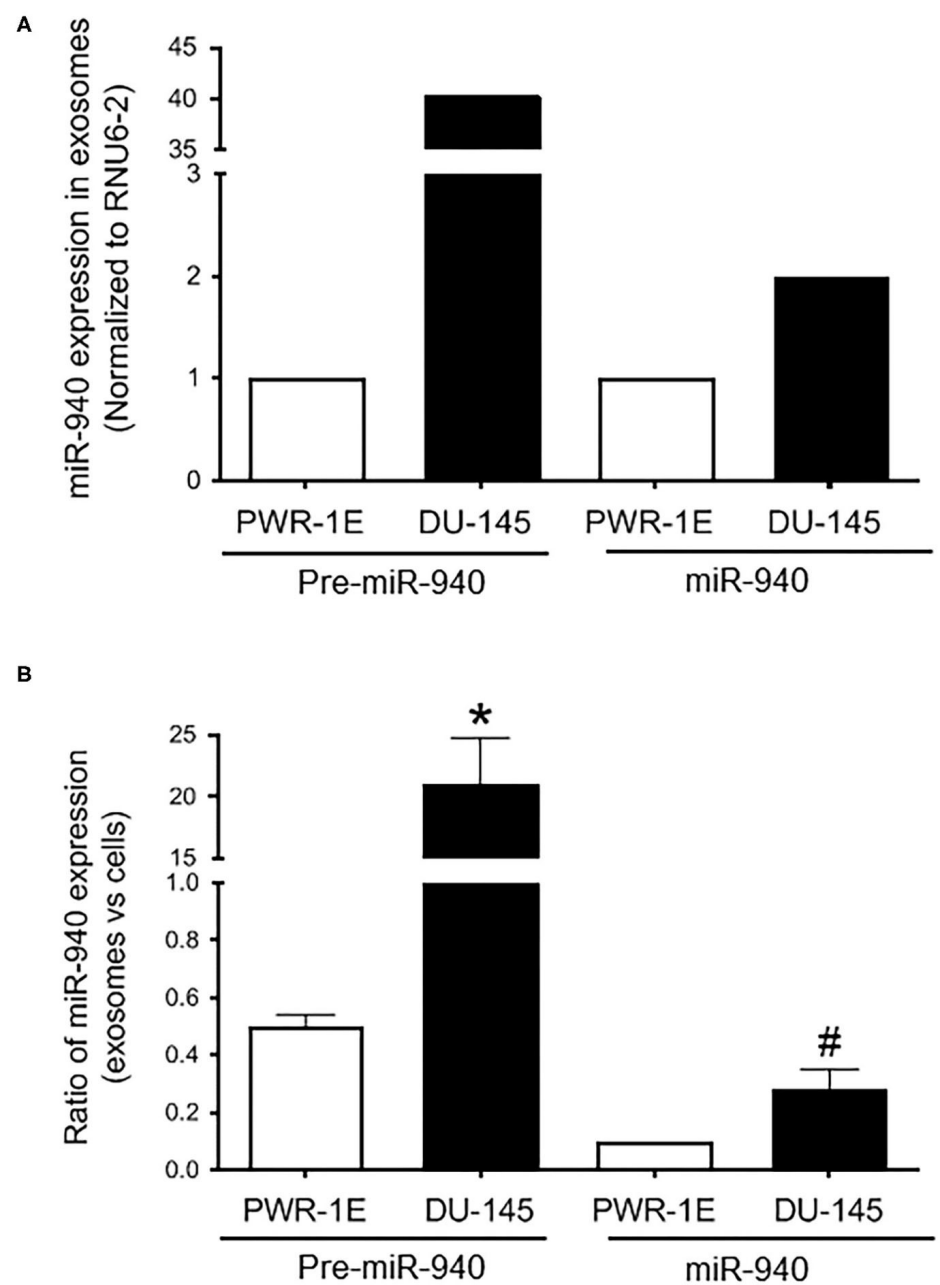
Expression of miR-940 in cell culture supernatant. **(A)** Higher levels of both precursor (Pre-miR-940) and mature (miR-940) miRNA are detected in the exosomes derived from the cell culture supernatant of the DU-145 cancer cells compared to the normal PWR-1E cells as measured by qPCR (normalized to RNU6-2). **(B)** Ratio of miR-940 from the secreted exosomes to miR-940 within the cells, as determined by qPCR, is plotted as a fold change in the normal and cancer cells. The experiment was repeated two independent times. ^#^*p-*value ≤ 0.1; *p-value ≤ 0.05.

We have previously demonstrated the role of miR-940 in prostate cancer progression *via* the regulation of MIEN1 (32). Here, we hypothesized that miR-940 is a secretory miRNA that could be used as a non-invasive molecular marker for a positive diagnosis of prostate cancer. To test this, we evaluated the expression of miR-940 in the exosomes derived from the serum/supplement free cell culture supernatants of PWR-1E and DU-145 cells by qPCR. Higher levels of both the precursor (Pre-miR-940) and mature (miR-940) miRNA were detected in the exosomes derived from the DU-145 cancer cells compared to the PWR-1E immortalized normal cells (Figure 1A). This was in stark contrast to the lower expression of miR-940 in DU-145 cell lysate compared to PWR-1E cell lysate as reported in our previous study (32). Since, the expression of miRNAs detected in both cell lines were isolated from the serum/supplement free cell culture supernatants, the observed differences are biologically relevant and not an artifact arising from intrinsic differences in the growth media for the two cell lines. Next, we quantified the proportion of miR-940 released in the exosomes to their levels within the cells for both the cell types. The ratio indicated that a higher proportion of miR-940 was secreted into the supernatant from DU-145 cells (Pre-miR-940: ~40-fold more; miR-940: ~3-fold more) than from PWR-1E (Figure 1B). This inverse expression pattern of miR-940 within and outside the cells suggested that miR-940 could be secreted out of the cells during cancer progression, in order to prevent the function of miR-940 mediated suppression of cancer, similar to what has been reported in ovarian cancer (48).

### Circulating miR-940 Is High in Serum Samples of Prostate Cancer Patients

**Figure 2:**
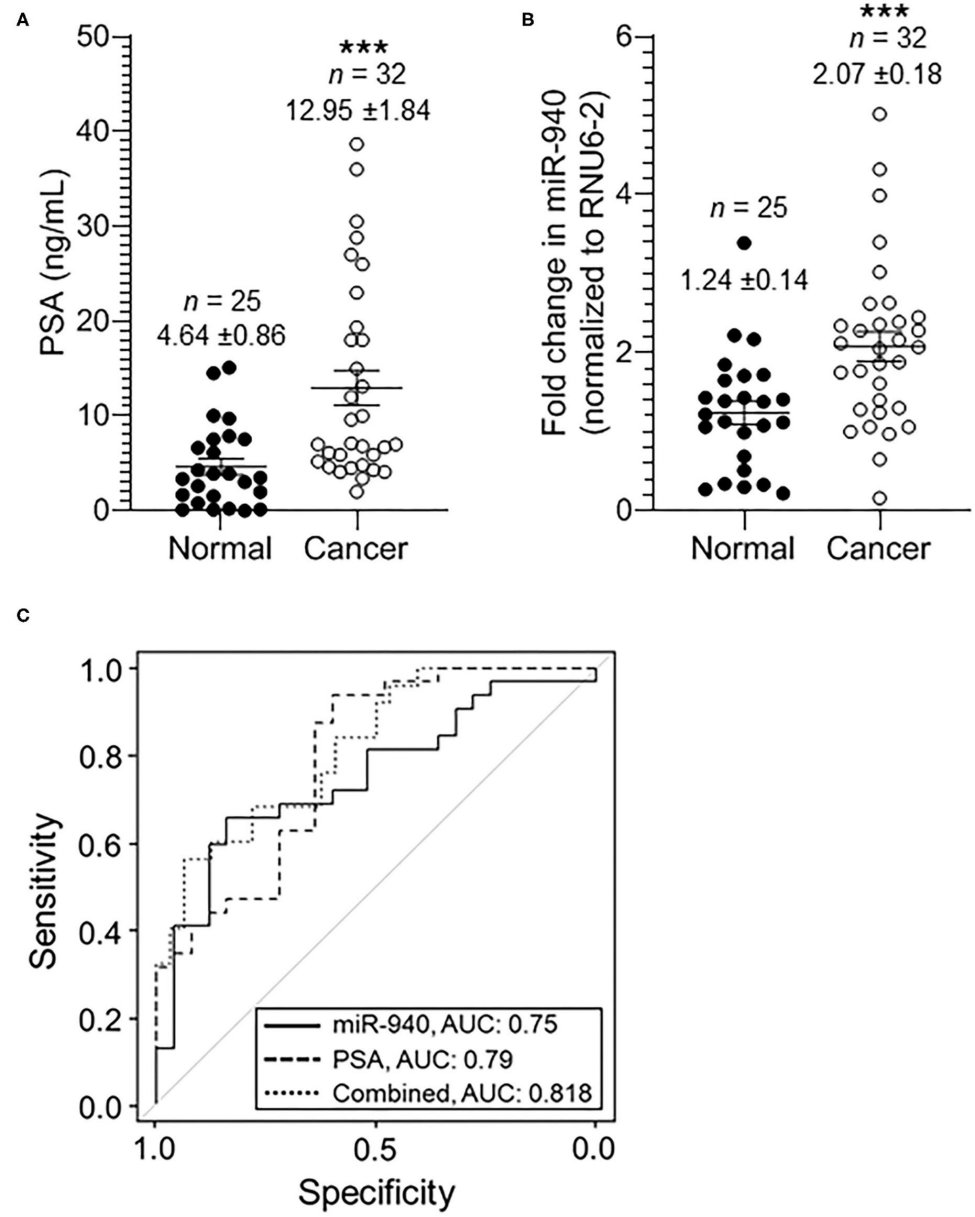
The expression of miR-940 and PSA in serum samples of prostate cancer patients and normal healthy males. **(A)** Scatter plot analysis of PSA expression in serum samples of normal healthy (*n* = 25) controls and prostate cancer (*n* = 32) patients. The data are expressed as the mean ± SEM (***p-value = 0.0005; two-tailed Student's *t*-test). **(B)** Scatter plot analysis of miR-940-fold expression in serum samples of healthy normal controls (*n* = 25) and prostate cancer patients (*n* = 32). The data are expressed as the mean ± SEM (***p-value = 0.0013; two-tailed Student's *t*-test). **(C)** ROC curve for **(B)** depicts the sensitivity (true positive: cancer in reality and according to test variable; i.e., fold change in miR-940) and specificity (100%—false positive: 100%—normal in reality with fold change in miR-940 predicting cancer) for the use of secreted miR-940 as an independent variable to determine cancer vs. normal states. The AUC values are shown on the graph.

The expression of miR-940 was next assessed in a cohort of serum samples obtained from healthy or cancer patients with varying PSA levels: 0.01–15.10 ng/mL in normal samples and 2.00–38.67 ng/mL in cancer serum. As shown in Figure 2A, the mean concentration of serum PSA levels in normal controls was 4.64 ± 0.86 ng/mL (*n* = 25), whereas that in prostate cancer patients was 12.95 ± 1.84 ng/mL (*n* = 32, *p*-value = 0.0002). Similarly, the levels of miR-940 were significantly higher in serum from cancer (*n* = 32, 2.07 ± 0.18 fold, *p-*value = 0.0008) patients compared to the normal (*n* = 25, 1.24 ± 0.14 fold) serum (Figure 2B). The area under the curve (AUC) for a receiver operating characteristic (ROC) curve determining the sensitivity and specificity of the test with miR-940 in serum as the disease indicator, is 0.75 [Figure 2C; 95% confidence interval (CI) = 0.622 to 0.877, *p*-value = 0.0013] and for PSA is 0.79 (95% CI = 0.670–0.909, *p*-value = 0.0002). The combination of the conventional prostate cancer serum biomarker of PSA and miR-940 generated an increased diagnostic AUC value of 0.818 (95% CI = 0.710–0.925), which is better than the PSA or miR-940 AUC alone. We have also compared model fits using logistic regression (PSA only vs. PSA + miR-940 as predictors). Under the null hypothesis of no improvement by adding miR-940 as a predictor, the *p-*value is 0.03, indicating statistical significance. Additionally, Spearman correlation coefficient between miR-940 fold-change and PSA is 0.3914 (0.1379–0.5967) with a significance of 0.0026, indicating that the use of miR-940 along with PSA is likely to improve the diagnostic accuracy of prostate cancer detection.

### Serum miR-940 Can Potentially Distinguish Well-Differentiated From Poorly Differentiated Tumors

**Figure 3:**
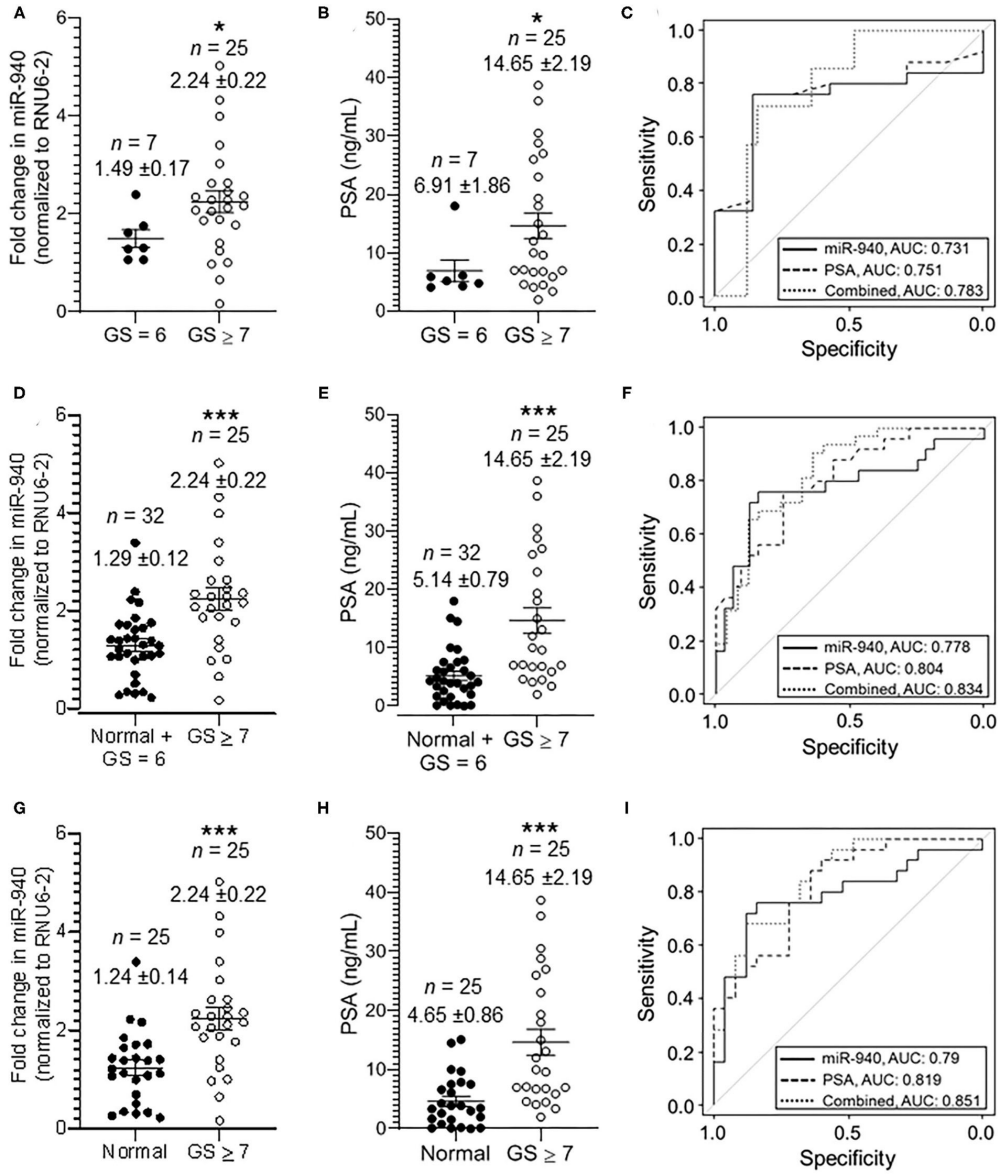
Secreted miR-940 and PSA from serum is significantly higher in patients with clinically significant tumors. **(A)** Serum from patients who had GS 7 and above in their biopsy were grouped together as clinically significant tumor serum (GS ≥ 7, *n* = 25) and the ones with biopsy GS 6 as clinically insignificant tumor serum (GS = 6, *n* = 7). The fold change in the miR-940 was quantified using qPCR. The data are expressed as the mean ± SEM (*p-value = 0.0148; two-tailed Student's *t*-test). **(B)** Scatter plot analysis of PSA expression in serum samples of clinically insignificant (*n* = 25) and clinically significant (*n* = 32) prostate cancer patients. The data are expressed as the mean ± SEM (*p-value = 0.0134; two-tailed Student's *t*-test). **(C)** ROC curve for both miR-940, PSA, and combined (miR-940 and PSA) comparing clinically insignificant to clinically significant tumors. **(D)** Scatter plot analysis of miR-940 expression in serum samples of combined normal and clinically insignificant (Normal + GS 6, *n* = 32) tumors and clinically significant tumors (GS ≥ 7, *n* = 25) patients. The data are expressed as the mean ± SEM (***p-value = 0.0006; two-tailed Student's *t*-test). **(E)** PSA expression in serum samples of combined normal and clinically insignificant (Normal + GS 6, *n* = 32) tumors and clinically significant (GS ≥ 7, *n* = 25) tumors patients. (***p-value = 0.0003; two-tailed Student's *t*-test) **(F)** ROC curve for fold change in miR-940 to determine clinically significant cancers (GS ≥ 7) vs. normal and clinically insignificant cancers (healthy controls + GS = 6). **(G)** Scatter plot analysis of miR-940 expression in serum samples of normal (*n* = 25) healthy controls and clinically significant tumors (GS ≥ 7, *n* = 25) patients. The data are expressed as the mean ± SEM (***p-value = 0.0005; two-tailed Student's *t*-test). **(H)** PSA expression in serum samples of normal (*n* = 25) controls and clinically significant tumors (GS ≥ 7, *n* = 25) patients. (***p-value = 0.0002; two-tailed Student's *t*-test) **(I)** ROC curve for fold change in miR-940 to determine clinically significant cancers (GS ≥ 7) vs. normal (healthy controls).

We classified the tumor serum further into moderately-poorly differentiated clinically significant (GS ≥ 7; PSA 2.0–38.67 ng/mL) and well-differentiated clinically insignificant (GS = 6; PSA 4.1–18.0 ng/mL) tumors, based on the pathological Gleason scoring rendered to the tumors during biopsy. There was an overall significant increase in miR-940 in moderately and poorly differentiated (*n* = 25, 2.24 ± 0.22 fold, *p*-value = 0.0148) tumor serum samples compared to clinically insignificant (*n* = 7, 1.49 ± 0.17-fold) tumors (Figure 3A). Similarly, the mean concentration of serum PSA levels in clinically insignificant tumors was 6.91 ± 1.86 ng/mL (*n* = 7), whereas that in clinically significant tumors was 14.65 ± 2.19 ng/mL (*n* = 25, *p*-value = 0.0134; Figure 3B). The degree of prediction using miR-940, in these well-differentiated and poorly differentiated samples, was however not better than the prediction based on PSA (Figure 3C). AUC for miR-940 ROC curve is 0.731 (95% CI = 0.541–0.921, *p-*value = 0.0649), while AUC for PSA ROC curve is 0.751 (95% CI = 0.566–0.936, *p*-value = 0.0449) and the combined (miR-940 + PSA) AUC is 0.783 (95% CI = 0.610–0.956). In this case, the improvement in model fit for logistic regression is not statistically significant (*p-*value = 0.18). The AUC for the ROC curve, however, increased to 0.778 (95% CI = 0.645–0.909, *p*-value = 0.0004) from the previously observed 0.75 (Figure 2C), when we combined the normal and clinically insignificant tumors (Normal + GS 6, *n* = 32, 1.29 ± 0.12-fold) into one category and compared them to clinically significant tumors (GS ≥ 7, *n* = 25, 2.24 ± 0.22-fold, *p-*value = 0.0006) for miR-940 (Figures 3D,F). In addition, AUC for PSA ROC curve was found to be 0.804 (95% CI = 0.693–0.916; *p-*value = 0.0001), when we combined the normal and clinically insignificant tumors (Normal + GS 6, *n* = 32, 5.14 ± 0.79 ng/mL) and compared them to clinically significant tumors (GS ≥ 7, *n* = 25, 14.65 ± 2.19 ng/mL, *p-*value = 0.0003) for PSA alone (Figure 3E), and similarly increased to 0.834 (95% CI = 0.726–0.942) for the combined (miR-940 + PSA) ROC curve (Figures 3E,F). In this case, the improvement in model fit for logistic regression is found to be statistically significant (*p-*value = 0.01). Comparing the normal miR-940 expression levels (*n* = 25, 1.24 ± 0.14-fold) to well-differentiated tumor serum (GS = 6, *n* = 7, 1.49 ± 0.17-fold) samples did not yield any significant difference (*p-*value = 0.2931). So, we next excluded the clinically insignificant tumor samples from the analysis to test the diagnostic accuracy of miR-940 levels between normal and clinically significant tumors. We observed that the miR-940 levels in serum from moderately-poorly differentiated tumors were significantly elevated (GS ≥ 7, *n* = 25, 2.24 ± 0.22 fold, *p-*value = 0.0005) compared to their levels in normal serum (*n* = 25, 1.24 ± 0.14-fold, Figure 3G), leading to a further improved AUC of 0.79 (Figure 3I; 95% CI = 0.658–0.922, *p-*value = 0.0004) for miR-940. Similarly, PSA levels were also significantly elevated in serum samples of clinically significant tumors (GS ≥ 7, *n* = 25, 14.65 ± 2.19 ng/mL, *p-*value = 0.0001) compared to normal sera (*n* = 25, 4.65 ± 0.86 ng/mL; Figure 3H). The AUC value was increased to 0.819 (95% CI = 0.705–0.933, *p-*value = 0.0001) for PSA, while improving the combined miR-940 and PSA value to 0.851 (95% CI = 0.748–0.955, *p*-value = 0.02; Figure 3I). With the use of a logistic regression model, we found that the combination of miR-940 along with PSA are able to reliably discriminate the clinically significant samples and healthy control samples, and are slightly better than PSA alone.

### Circulating miR-940 Is Detected in Urine Samples of Prostate Cancer Patients

**Figure 4:**
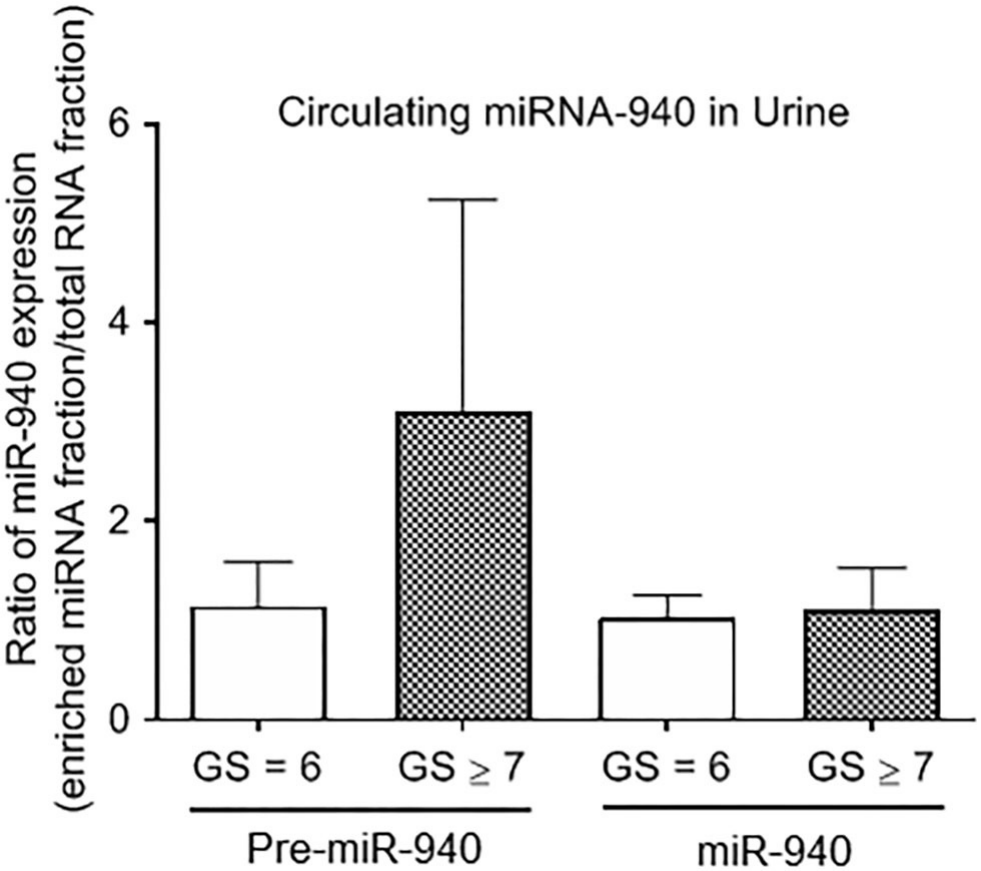
The expression of miR-940 in urine of prostate cancer patients. Relative expression of Pre-miR-940 and miR-940 in the urine from clinically insignificant (GS ≥ 7) prostate cancer patients relative to clinically insignificant (GS = 6) patients (*n* = 3 and 5 respectively).

In this pilot study, we also quantified both precursor and mature miR-940 in the urine sample of prostate cancer patients. Although there were significantly lower amounts of RNA detected in the urine compared to the blood in general, we were still able to detect traces of miR-940, but this was not significantly different between clinically insignificant and clinically significant tumors (Figure 4; Pre-miR-940: 1.15 ± 0.45-fold and 3.11 ± 2.13-fold; miR-940: 1.04 ± 0.21-fold and 1.12 ± 0.42-fold from clinically insignificant and significant tumors, respectively).

## Discussion

The current standard for prostate cancer detection includes DRE with PSA, followed by biopsies, for confirmation of the clinical diagnosis (4–6). With the relatively low diagnostic accuracy of PSA, and secondary infections associated with biopsy, there has been a clinical deadlock in prostate cancer diagnosis. To overcome this predicament, extensive research is underway to find reliable non-invasive markers which will not only increase the precision of diagnosis but also reduce patient discomfort. This study presents the possible use of circulating miR-940 as a secondary diagnostic biomarker for prostate cancer (in addition to the PSA). In this pilot study we have shown that miR-940 levels are high in serum from cancer patients compared to healthy controls. Although, we have primarily used equal amount of total RNA from different serum samples to detect miR-940 levels, we have also normalized to RNU6-2 (49, 50). Given that RNU6-2 is not a miRNA itself and could be released and expressed at different levels between normal and cancer patients, based on the results, we could also speculate that the miR-940/RNU6-2 combination in cancer serum is higher than in normal serum. We acknowledge that our study also has some limitations including the relatively small sample sizes and the lack of follow up information of the patients. Also, although some of our healthy controls had high PSA with no history of prostate cancer diagnosis, they did not have a biopsy. Hence, further studies with patients who demonstrate elevated PSA but a negative biopsy and/or more samples with normal PSA as the healthy control group are needed to obtain a better diagnostic value for miR-940 by itself. Despite these drawbacks, the strong correlation of PSA to circulating miR-940 in the serum still suggests potential improvement in diagnostic accuracy for prostate cancer.

Our previous study has shown miR-940 to be highly expressed in normal cells and tissues compared to cancer counterparts (32). The expression of miR-940 in the serum is contradictory to this intracellular pattern as observed in a study conducted in breast cancer where 28 miRNAs were opposing in terms of their expression patterns between the tissue and the serum (51). This may be attributed to the many ways miRNAs are trafficked between cells or exported out of the cells, if their intracellular targets are oncogenes or tumor suppressors or to facilitate cell-cell communication (52–54). There are contradicting evidences for the presence of miR-940 in the serum: while some did not observe detectable levels of miR-940 (25), other detected miR-940 in circulation (55–57). Although we assume that the miR-940 detected in the serum is primarily from the prostate cells, we cannot with absolute certainty exclude the miRNA secretion from other cells. However, since the primary difference between the cohorts of healthy or prostate cancer patient samples is the prostate cancer disease status, we assume that at least a larger part of the secreted miRNA could be associated with the cancer cells.

Here, we speculate a bimodal role for miR-940 that could explain its increase in serum from cancer patients: (1) miR-940 is exported out of the cancer cells to prevent the down regulation of MIEN1, and other proteins predicted as targets inside the cell that aid in cancer progression (32–34, 36, 58) and (2) the secreted miR-940 facilitates the microenvironment remodeling to allow cancer progression (40, 59).

In the recent years, a few studies have identified the contradictory roles of circulating miR-940 in various cancers, either its increase or decrease is associated with cancer diagnosis (25, 33, 40, 55–57, 59, 60). Since the origin of the circulating miRNAs in serum cannot be identified, the use of just miR-940 in the serum has to be made with utmost caution and supported by other tests in order to maximize its use as a diagnostic biomarker. Here, even though we show that the miR-940 levels by themselves can distinguish cancer from normal, we still use PSA as a supportive diagnostic variable and suggest that the use of miR-940 with PSA will be more powerful than using PSA alone for the diagnosis of prostate cancer. Considering that this study strongly alludes to miR-940 being a biomarker for prostate cancer, larger scale validations are warranted.

## Data Availability Statement

The raw data supporting the conclusions of this article will be made available by the authors, without undue reservation.

## Ethics Statement

The studies involving human participants were reviewed and approved by Institutional Review Boards of the University of North Texas Health Science Center, Fort Worth, TX (IRB # 2007-110, and 2013-016) and the University of Texas Southwestern Medical Center, Dallas, TX (IRB # STU 032011-187). The patients/participants provided their written informed consent to participate in this study.

## Author Contributions

SR, JV, and PC conceived, designed the study, and wrote the paper. SR performed all the experiments, except for exosome isolation, and analyzed the data. SM performed exosome isolations from cell culture supernatant. YL, JV, and PC collected most of the serum and urine samples. SR, AH, YL, RN, JV, and PC interpreted the results. All authors contributed to the article and approved the submitted version.

## Conflict of Interest

The authors declare that the research was conducted in the absence of any commercial or financial relationships that could be construed as a potential conflict of interest.
